# ASSOCIATIONS OF HOME ENVIRONMENT AND UNMET CARE NEEDS AMONG LOW- AND MODERATE-INCOME OLDER ADULTS

**DOI:** 10.1093/geroni/igad104.2010

**Published:** 2023-12-21

**Authors:** Safiyyah Okoye, Laura Samuel, Chanee Fabius, Craig Pollack, Laura Gitlin, Sarah L Szanton, Jennifer Wolff

**Affiliations:** College of Nursing and Health Professions, Drexel University, Philadelphia, Pennsylvania, United States; Johns Hopkins University School of Nursing, Baltimore, Maryland, United States; Johns Hopkins University, Baltimore, Maryland, United States; Johns Hopkins, Baltimore, Maryland, United States; Drexel University, Philadelphia, Pennsylvania, United States; Johns Hopkins University, Baltimore, Maryland, United States; Johns Hopkins University, Baltimore, Maryland, United States

## Abstract

The home environment is an increasingly important setting for long-term services and support. However, more information is needed about the relationship between the home environment and adverse consequences due to unmet needs (i.e., going without assistance with everyday activities such as eating or bathing) among older adults. We draw on information from N=4,898 low and moderate-income (annual income <$60,000) community-living respondents to the National Health and Aging Trends Study (NHATS). Two aspects of the home environment were directly observed by NHATS interviewers: home disorder (six items, including flooring problems, tripping hazards, pests) and home disrepair (five items, including missing siding and broken steps). NHATS asked participants who reported a disability whether they experienced one of seven adverse consequences (e.g., going without eating) due to no one being there to provide help or the activity is too difficult for them to complete on their own. About half of our sample (48.5%) reported a disability. We found home disorder and disrepair more prevalent among older adults with disabilities than those without (36.2% v. 28.1% and 18.2% v. 15.0%, respectively). Among older adults with disabilities, the presence of any home disorder and any home disrepair were associated with higher odds of experiencing an adverse consequence of an unmet care need in unadjusted analyses (home disorder: OR 1.33; 95% CI 1.11, 1.60; home disrepair: OR 1.16; 95% CI 0.90, 1.50). Findings show an association between home environment and unmet care needs and can signal the need to target housing-related risk factors in future interventions.

